# Molecular Mimicry of the Viral Spike in the SARS-CoV-2 Vaccine Possibly Triggers Transient Dysregulation of ACE2, Leading to Vascular and Coagulation Dysfunction Similar to SARS-CoV-2 Infection

**DOI:** 10.3390/v15051045

**Published:** 2023-04-25

**Authors:** Christian A. Devaux, Laurence Camoin-Jau

**Affiliations:** 1 Microbes Evolution Phylogeny and Infection (MEPHI) Laboratory, Aix-Marseille University, Institut de Recherche Pour le Développement (IRD), Assistance Publique Hôpitaux de Marseille (APHM), Institut Hospitalo-Universitaire (IHU)–Méditerranée Infection, 13005 Marseille, France; laurence.camoin@ap-hm.fr; 2 Centre National de la Recherche Scientifique (CNRS-SNC5039), 13000 Marseille, France; 3 Laboratoire d’Hématologie, Hôpital de La Timone, APHM, Boulevard Jean-Moulin, 13005 Marseille, France

**Keywords:** ACE2, renin-angiotensin system, hypertension, coagulation, coronavirus, COVID-19, therapy

## Abstract

The benefits of SARS-CoV-2 spike mRNA vaccines are well known, including a significant decline in COVID-19 morbidity and a decrease in the mortality rate of SARS-CoV-2 infected persons. However, pharmacovigilance studies have revealed the existence of rare cases of cardiovascular complications after mass vaccination using such formulations. Cases of high blood pressure have also been reported but were rarely documented under perfectly controlled medical supervision. The press release of these warning signals triggered a huge debate over COVID-19 vaccines’ safety. Thereby, our attention was quickly focused on issues involving the risk of myocarditis, acute coronary syndrome, hypertension and thrombosis. Rare cases of undesirable post-vaccine pathophysiological phenomena should question us, especially when they occur in young subjects. They are more likely to occur with inappropriate use of mRNA vaccine (e.g., at the time when the immune response is already very active during a low-noise infection in the process of healing), leading to angiotensin II (Ang II) induced inflammation triggering tissue damage. Such harmful effects observed after the COVID-19 vaccine evoke a possible molecular mimicry of the viral spike transiently dysregulating angiotensin converting enzyme 2 (ACE2) function. Although the benefit/risk ratio of SARS-CoV-2 spike mRNA vaccine is very favorable, it seems reasonable to suggest medical surveillance to patients with a history of cardiovascular diseases who receive the COVID-19 vaccine.

## 1. Introduction

Production of an effective vaccine has always been the best possible method of protecting a population against infectious agents, which represent a public health problem. Once this basic principle is accepted, the question that arises is the best design of the vaccine (with or without adjuvant such as aluminium salts) to be chosen from a large panel of solutions, including the traditional live attenuated virus (such as the poliovirus vaccine); the inactivated virus (by chemical or physical treatments); the more sophisticated approach of a viral vector vaccine expressing the recombinant envelope protein of the agent to be combated as a result of advances in biotechnology; protein-based vaccines (such as recombinant envelope proteins or epitope-based synthetic polypeptides), which are delivered by polymers or liposomes microparticles and, more recently, the development of an RNA vaccine (RNA coding for the viral envelope protein), a strategy which makes it possible to encode a viral protein [1,2]. Each method has its advantages and disadvantages, and in each case the benefit/risk ratio must be assessed. Live virus vaccines have the capacity to infect cells and generate infectious virions as in a natural infection, but there is a major risk of inducing a disease, even with the attenuated virus vaccine (the virus can sometimes revert to the wild type genotype). Inactivated viruses expose the viral proteins to the immune system, but there is no virus replication. Recombinant envelope proteins or synthetic polypeptides may provide an efficient way of inducing neutralizing antibodies capable of blocking free viral particle entry into cells, but are poorly effective against cell-associated viruses. An efficient immune response may require the induction of CD8+ cytotoxic T lymphocytes, which target virally infected cells but not free virus particles.

**Figure 1:**
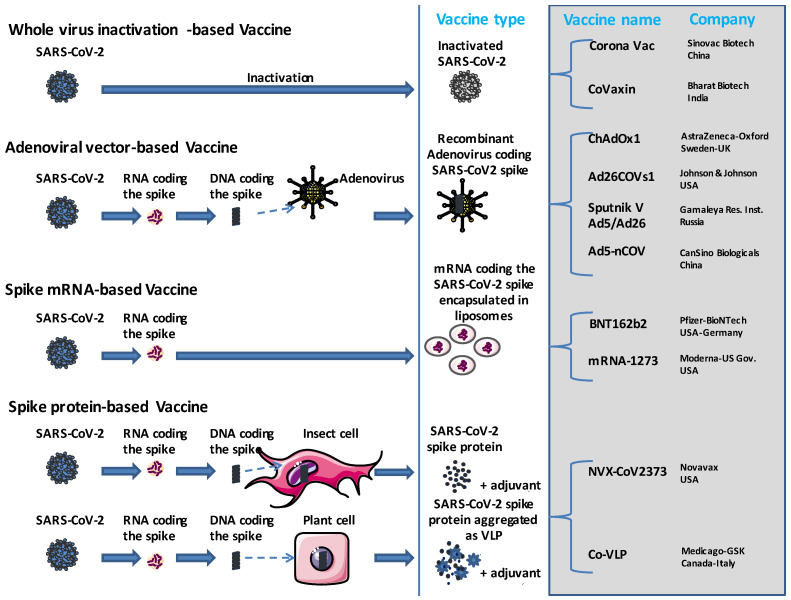
The main strategies used for the design of the SARS-CoV-2 vaccine. COVID-19 vaccines are based on either: (i) whole inactivated SARS-CoV-2 particles (this is the most traditional technology that uses a non-replicative SARS-CoV-2 vaccine, the advantage of which resides in the fact that all viral antigenic proteins are available to prime the host immune system), (ii) an adenoviral vector (the DNA sequence coding the SARS-CoV-2 spike is carried by a replicative recombinant adenovirus that acts as a viral shuttle), (iii) the recent technology of mRNA-based vaccines (the SARS-CoV-2 spike mRNA requires special packaging such as liposomes to allow it to be stably delivered to humans by injection, where it enters human cells that temporarily produce the SARS-CoV-2 spike protein) and (iv) the spike protein-based technology that exploits transgenic cells as producers of SARS-CoV-2 spike protein. Of course with all the recent developments in biotechnology, there is a large panel of possible strategies, some of which have led to interesting results, as shown with the Abdala/CIGB-66 vaccine developed by the Center for Genetic Engineering and Biotechnology in Cuba based on the expression of a chimeric SARS-CoV-2 spike RBD (C-RBD-H6-PP) in Pichia pastoris yeast. VLP = virus like particle.

The SARS-CoV-2 spike (S) protein is the main target of both B-cells (which produce neutralizing antibodies) and cytotoxic T-cells (aimed at killing SARS-CoV-2-infected target cells) immune responses. Since the onset of COVID-19 [3,4,5], the vaccine strategies that have been introduced to protect patients against severe forms of COVID-19 include mRNA-based technology, recombinant DNA (full-length SARS-CoV-2 spike), viral vector (full-length S or S1 spike subunit inserted in modified adenovirus vector or vaccinia vector), viral subunits (full-length S, S1, S receptor binding domain known as RBD sequence and nucleocapsid), virus-like particles (S, S1, RBD, or co-expression of S1, M and E proteins produced in baculovirus) and protein or peptide vaccines [6,7]. The mRNA vaccines gained considerable attention at the beginning of the COVID-19 pandemic, mainly because they were found to be easy to design (and presented great flexibility in term of manipulating the coding sequence and the possibility of simultaneously containing multiple mRNAs in a single dose of vaccine). They also involved short development and production cycles, and could be produced at a low cost. The novel mRNA therapeutic tools that encode the antigen of interest (e.g., SARS-CoV-2 spike), can be non-replicating mRNA (in vitro transcribed mRNA) containing 5′ and 3′ untranslated regions or self-amplifying mRNA (self-replicating mRNA/replicon), which, in addition to the antigen of interest, also encode the viral replication machinery, leading to high intracellular RNA amplification and abundant protein synthesis. The route of injection is likely to be important. For instance, intramuscular injection (the administration route of the mRNA-based vaccine) mainly induces protective IgG responses but has no considerable effect on lung mucosal immunity, including the specific IgA secretion and stimulation of local memory T cells [8,9]. In the race to develop an anti-SARS-CoV-2 vaccine, the pharmaceutical companies opted for different strategies. The Oxford–AstraZeneca and Johnson and Johnson formulations contain DNA delivered within non-replicating recombinant adenovirus vector-based systems, while both the Pfizer and Moderna vaccine formulations utilize the novel mRNA technology combined with lipid nanoparticle delivery systems. However, others such as the Sinovac vaccine use more traditional strategies [10] (Figure 1).

With the objective of preventing viral spread in humans, limiting the pandemic and reducing the frequency of severe forms of COVID-19 (decline in COVID-19 morbidity and decrease in the mortality rate of SARS-CoV-2 infected persons), the World Health Organization (WHO) approved the safety and efficacy of several anti-COVID-19 vaccines including the Oxford–AstraZeneca, Johnson and Johnson, Pfizer-BioNTech, and Moderna products. Billions of doses of SARS-CoV-2 mRNA vaccines were thus distributed to and injected in people worldwide. With the documentation of the mRNA-based SARS-CoV-2 spike vaccine, side effects not previously recognized began to be documented, including myocarditis, hypertension, thrombosis and thromboembolic risks, as well as acute coronary syndrome. Rare thrombocytopenia and thromboembolic events after injection of the ChAdOx1 nCov-19 (Oxford–AstraZeneca) vaccine were documented [11,12,13,14]. Conversely, the BNT162b2 mRNA (Pfizer-BioNTech) vaccination was associated with arterial thromboembolism 15–21 days after vaccination, but not with thrombocytopenia [15]. These adverse pathophysiological effects have also been observed with natural SARS-CoV-2 infection. This should draw our attention to the nature of the vaccine and the S protein that binds to the ACE2 receptor [16,17]. ACE2 should not be seen as a simple viral receptor, since ACE2 plays a major role in the regulation of blood pressure (BP) and in coagulation [18,19]. It is currently admitted that the SARS-CoV-2 spike, likely at high concentrations, could be the first cause of the pathological phenomena described with severe COVID-19. This leads us to put forward the hypothesis that in rare cases, the antigenic mimicry sought with the SARS-CoV-2 spike vaccination could have a side effect on the receptor, predisposing certain vaccinated individuals to experience coagulation disorders shortly after injection of the vaccine. If this hypothesis is confirmed, then a short course of anticoagulant treatment would be likely to be beneficial to protect vaccinated people against these adverse effects.

## 2. Myocarditis and Acute Coronary Syndrome after COVID-19 Vaccine

Rare and sometimes fatal cardiovascular complications have been reported after mass vaccination [20,21,22]. These findings paved the way for a debate over adverse outcomes and issues involving cardiovascular health following COVID-19 vaccination. Notably, the risk of thrombosis and myocarditis following immunization has particularly attracted the attention of caregivers and epidemiologists [23]. In late May 2021, the WHO vaccine safety committee noted that myocarditis and pericarditis following vaccination with COVID-19 mRNA vaccines required further investigation. Acute viral myocarditis is the inflammation of myocardium secondary to cytotoxicity resulting in myocyte degeneration and necrosis [24,25].

Before COVID-19, the incidence of myocarditis (commonly caused by viruses such as influenza, coxsackie, herpes, hepatitis or bacteria) was between 1/100,000 and 10/100,000 cases per year, with higher rates in young adult males. According to the US Centers for Diseases Control and Prevention (CDC), the risk of myocarditis after infection with COVID-19 is much higher at 146/100,000 cases per year. Based on a study on 304 people from Israel with symptoms of myocarditis following administration of the BNT162b2 vaccine, there was an increased risk in young adult males after the second vaccine dose [26]. Although one fulminant and fatal case did occur, symptoms were mild in 95% of recipients. A report from Denmark [27] reviewed data from five million residents aged twelve and older, four million of whom had received one of the mRNA vaccines (BNT162b2 or mRNA-1273). Their results confirmed that although rare, post-vaccine myocarditis does exist, with an incidence of 1.4/100,000 vaccinated individuals. With the BNT162b2 vaccine, post-vaccine myocarditis was associated mainly with females. With the mRNA-1273 vaccine, myocarditis or myopericarditis (76% male, 86% second dose) was reported with an overall incidence of 4.2/100,000. The risk was increased in both male and female participants and was substantially higher after the second dose. Another investigation [28] into hospital admissions and deaths from myocarditis, pericarditis and cardiac arrhythmias following administration of an adenovirus (ChAdOx1, *n*  =  20,615,911) or messenger RNA-based vaccine (BNT162b2, *n*  =  16,993,389; mRNA-1273, *n*  =  1,006,191) highlighted an increased risk of myocarditis associated with the first dose of ChAdOx1 and BNT162b2 vaccines and the first and second doses of the mRNA-1273 vaccine over the 1–28 day post-vaccination period. This is estimated as an extra 0.2, 0.1 and 0.6 myocarditis events per 100,000 people vaccinated with ChAdOx1, BNT162b2 and mRNA-1273, respectively. This figure should be compared with an extra four myocarditis events per 100,000 patients in the 28 days following a SARS-CoV-2 positive test. Increased risk of cardiac arrhythmias was also found after a second dose of mRNA-1273. The overall incidence of post-vaccine myocarditis is currently estimated between 0.3 and 5 cases per 100,000 people. The highest incidence of myocarditis after vaccination with mRNA vaccines has occurred within three to four days after the second vaccination in males under 30 years of age. A recent study [29] analyzed 160 patients with carditis and 1533 without, linking health care records to vaccination records. The BNT162b2 mRNA vaccine was found to be associated with 20 cases of carditis, while CoronaVac, an inactivated virus vaccine, was found to be associated with seven carditis cases. The authors concluded that patients who were vaccinated with BNT162b2 were found to be three times more likely to have carditis than unvaccinated participants; however, no elevated risk was found for CoronaVac. The increased risk was seen predominantly among males and was more likely to be seen in adolescents, after the second dose of BNT162b2 rather than the first. Although they are rare events, carditis and multisystem inflammatory syndrome (MIS) can sometimes be associated with COVID-19 vaccine, especially in young subjects whose antiviral immunity is very powerful [30,31]. However, most studies conclude on the clear benefits of COVID-19 mRNA vaccination with respect to myocarditis.

Notably, these side effects are more likely to occur with inappropriate use of the mRNA vaccine (e.g., at the time when the immune response is already very active during a low-noise infection in the process of healing), leading to exacerbated cytotoxic T cell response triggering tissue damage [32,33,34]. mRNA COVID-19 vaccines dramatically increased endothelial inflammatory markers and acute coronary syndrome (ACS) risk [35]. In this study the author investigated the risk of ACS in 566 patients using the PULS Cardiac Test based on changes in IL-16 (proinflammatory cytokine), soluble FAS (inducer of apoptosis) and hepatocyte growth factor (chemotaxis of T cells). Dramatic changes in the PULS score (from 11% to 25%) were observed with patients immunized using the Moderna and Pfizer vaccines, and these changes persisted for at least 2.5 months after the second dose of vaccine. The author concluded that “the mRNA vacs dramatically increase inflammation on the endothelium and T cell infiltration of cardiac muscle and may account for the observations of increased thrombosis, cardiomyopathy, and other vascular events following vaccination”. Cases of multisystem inflammatory syndrome (MIS), which mainly affects children, following vaccination were reported [36,37], and were associated with elevated anti-AT1R, anti-endothelin receptor, anti-α1 adrenergic receptor, anti-β1 adrenergic receptor, anti-β2 adrenergic receptor and anti-muscarinic cholinergic receptor-2/3/4 auto-antibodies [38]. In addition a few studies have reported evidence of symptomatic hypertension (malaise, headache, tingling in the mouth, diaphoresis and increased BP) after vaccination with Pfizer-BioNTech [39,40]. The potential implication of lipid nanoparticles in the pathogenesis of myocarditis associated with mRNA vaccine has also been suggested [41].

## 3. Hypertension and COVID-19 Vaccine

**Figure 2:**
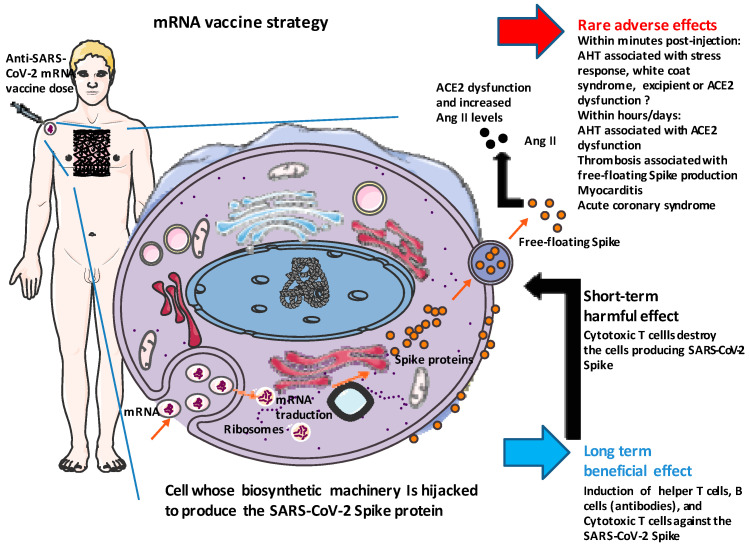
Schematic mechanism of action of an mRNA vaccine and potential interaction with the RAS cascade. The anti-SARS-CoV-2 mRNA vaccine formulation contains mRNA, which is able to encode the spike protein of SARS-CoV-2. The S mRNA requires special packaging to be stably delivered to human by injection and to enter target cells. Once inside cells, this mRNA forms a complex with initiation factors and the small subunit of the ribosome, where elongation of the polypeptide chain starts. During this period, the biosynthetic machinery of the cell is diverted to temporarily synthesize the SARS-CoV-2 spike protein. The S protein first assembles to form homotrimers into the cytoplasm and then migrates to the cell surface to protrude with a native-like conformation. These spike proteins trigger an anti-SARS-CoV-2 S protein immune response beneficial for the vaccinated individual. However, cells expressing the S protein can also be destroyed by a specific anti-S immune response. A free floating spike can be released, leading to a massive interaction with ACE2.

On 11 February 2021 the French Agency for the Safety of Medicines and Health Products (ANSM), responsible for monitoring vaccines against COVID-19, reported the occurrence of 73 cases of arterial hypertension (AHT) after injection of the tozinameran Comirnaty^®®^ vaccine from Pfizer-BioNTech (BNT162b2) (ANSM, 4th pharmacovigilance report; https://ansm.sante.fr/actualites/point-de-situation-sur-la-surveillance-des-vaccins-contre-la-covid-19-6; accessed on 4 April 2023) These surprising cases of AHT occurred either within minutes of the injection or within a few days, and most often resulted in clinical signs such as malaise, headaches, dizziness and cardiac arrhythmias. Comirnaty’s pharmacovigilance report mentioned several post-vaccination deaths, which occurred in very old and frail people from Vigibase, the WHO pharmacovigilance database. Among 91,761 reports of adverse drug reactions involving COVID-19 vaccines, hypertension was reported in 1776 cases (1.9%), mainly with tozinameran Comirnaty^®®^ (*n* = 1325; 75%) [42]. Analysis of data from Web Reports of EudraVigilance of the European Medicines Agency (EMA), in June 2021, showed approximately 3% of AHT with less than 0.5% of grade III hypertension [43]. The analysis conducted by Kaur and colleagues [44] over a period between 15 December 2020 and 24 January 2021 in India identified 4863 cardiovascular adverse events including 5.8% of AHT in a cohort of 30,523 vaccinated subjects. In June 2021, Meylan and colleagues [39] reported nine cases of unexplained stage III AHT in patients after receiving an mRNA-based SARS-CoV-2 vaccination, of which eight were symptomatic. Two patterns were observed, either a sudden increase in BP within minutes of vaccination or an increase in BP in the days following the administration of the vaccine. Vital signs were measured with at least three sets of separate values at five-minute intervals. The median age was 73 years, and the sex distribution was seven women and two men. Eight of nine patients had a history of AHT, with most patients on antihypertensive therapy and with well-controlled AHT. All but one patient received the Pfizer-BioNTech (BNT162b2) vaccine. These authors excluded an RAS imbalance due to viral mimicry and concluded that “an interaction between the S-protein and ACE2 also seems highly unlikely as patients reacted within minutes of the injection, not leaving time for mRNA cellular uptake, translation, and S-protein presentation at the membrane of macrophages and dendritic cells”. This conclusion could be debated for immediate AHT reactions within minutes of vaccine injection, which has been considered too short for cellular uptake of mRNA, formation of the preinitiation complex with initiation factor (IF) proteins and binding to the small subunit of the ribosome, priming with tRNA and elongation of the polypeptide chain, protein maturation and membrane targeting, leading to SARS-CoV-2 spike interaction with ACE2. Thus, for the short adverse effects, mechanisms other than down regulation of ACE2 have been suggested, such as a stress response, white coat syndrome and the possible role of polyethylene glycol as an excipient [39,45]. However, the endocytosis of the mRNA formulation is expected to take about 1 min and the translation speed has been estimated at 10 amino acids per second (the synthesis of most protein molecules takes between 20 s and several minutes), so about two minutes for the spike (1273 amino acids) [46,47], while the time needed for this protein to interact with ACE2 and impair the enzymatic activity of ACE2 until an effect on BP is obtained remains to be estimated (Figure 2).

Although the RAS-independent model of post-vaccine stage III AHT observed within minutes of the injection can be debated, it no longer applies once the endogenous synthesis of the SARS-CoV-2 spike is fully efficient. Free-floating spike proteins produced by vaccination exhibit receptor-binding capacity, as is the case with the native SARS-CoV-2 S protein [48]. Thus it can bind to ACE2, the main function of which is to regulate the balance between Ang II and angiotensin-(1-7) [Ang-(1-7)]. A decrease in ACE2 enzymatic activity results in the imbalance of the Ang II/Ang-(1-7) ratio characterized by an excess of Ang II leading to inflammation, aldosterone and vasopressin release, renal sodium reabsorbption and fibrosis [19]. Taken together these physiological disorders could directly contribute to an excessive rise in BP following injection of vaccine, as well as in the acute phase of SARS-CoV-2 infection.

Zappa and colleagues [40] reported the results of a prospective survey among health workers (mean age 43 years), including 113 people who received two doses of the Pfizer-BioNTech (BNT162b2) vaccine at 21-day intervals. Of these, 87% reported adverse reactions after the first dose and 83% after the second dose of vaccine. Most reactions were mild, and none of the subjects discontinued normal daily activities. Tachycardia was reported by 13% of individuals, and a rise in AHT occurred in 5% of subjects during the first five days post-injection. Similar results were obtained by Tran and colleagues [49]. Since these early reported results, many clinicians from different countries have monitored and reported similar observations. A French retrospective analysis of 23,358 subjects (of whom 21,909 had complete data on BP) investigated post-injection AHT in subjects who had received at least one dose tozinameran Comirnaty^®®^ vaccine (Pfizer-BioNTech BNT162b2) [50]. Among the 21,909 subjects, 8121 people (37.1%) exhibited systolic BP and/or diastolic BP above 140 mmHg and/or 90 mmHg (high BP), respectively, after the first injection. Those with high BP after the first injection were older than those with normal BP (mean age 69 years compared to 53 years); 692 people (3.2%) presented with grade III hypertension after the first injection, and those subjects were older than those with normal BP (74 ± 13 versus 58 ± 20 years) (*p* < 0.001). Another report investigating post-injection adverse effects of anti-SARS-CoV-2 vaccination among a population of 1870 subjects (69% female, 31% male), who had mostly received a first dose or a second dose of Comirnaty^®®^ Pfizer, indicated that 153 subjects (8%) reported an increase in BP values after vaccination, and 70 subjects (4%) observed a decrease in blood pressure, with higher frequency of BP alterations at the second or booster dose [51]. Another study on a total of 797 healthcare workers participants (mean age 48.1 ± 10.8 years, 63% women, 39% smokers), reported a significant increase in BP after Pfizer-BioNTech BNT162b2 vaccination among healthcare workers [52]. Seven participants reported an increase in their BP (three in the range of grade 2 and four in the range of grade 3 hypertension), with only one participant with a history of treated hypertension. The BP increase was observed at the end of the first week after the first dose, lasted for three to four days and recurred promptly after the second dose. Only one case required hospitalization. A survey of BP immediately after and 15–30 min post-vaccination of individuals receiving a second dose of the tozinameran (BNT162b2) mRNA COVID-19 vaccine among staff individuals indicated a general increase in BP in more than half of the subjects. Among those who experienced adverse effects, 84.5% claimed the severity was worse for the second dose. The most common adverse effects were redness, pain or swelling at the injection site, tiredness, fever, chills, headache and myalgia. Overall, 58 subjects (1.02%) were admitted to the observation room either due to a hypertensive emergency or complaints of dizziness [53]. In a meta-analysis including 357,387 subjects, the prevalence of abnormalities or increases in BP was 3.2%, and that of stage III hypertension was 0.6%, mainly in elderly and frail subjects [54]. It concluded that, although low, these risks should be taken into account, and these increases in BP should not be considered as sporadic events. However, these studies should be interpreted with caution. Lockdowns led to a decrease in the medical follow-up of patients with AHT, but also to an increase in risk factors such as sedentary lifestyle and poor dietary habits. There are many differences among these studies, particularly in the time between measurement of BP and vaccination. Finally, most patients did not carry out regular BP monitoring before vaccination (Table 1).

## 4. Thromboembolic Events after COVID-19 Vaccine

According to the international COVID-19 vaccination dataset, 361,734,967 people received a vaccination between 13 December 2020 and 16 March 2021. The Vigibase (Uppsala, Sweden) reported 2061 thrombotic events by 16 March 2021, including 1197 persons who received the BNT162b2/Comirnaty^®®^ from Pfizer-BioNTech, 639 persons who received the ChAdOx1 nCoV-19/AZD1222 from Oxford–AstraZeneca, and 325 persons who received the mRNA-1273 vaccine from Moderna. The calculated rate of thrombotic cases in the vaccinated population was 0.021/100,000, and the rates were 0.0075/100,000 for venous thrombotic events and 0.013/100,000 for arterial thrombotic events and could likely occur with all three vaccines [55].

The Oxford–AstraZeneca COVID-19 vaccine, ChAdOx1 nCoV-19 adenovirus vaccine (AZD1222), was associated with a risk of vaccine-induced thrombosis in the range of 0.02–2 cases/100,000 vaccinations, the highest risk of thrombosis concerning younger women [56]. Similar thromboembolic events were reported in a study of patients who had received the Johnson & Johnson vaccine Ad26.COV2.S (a recombinant adenovirus serotype 26 vector encoding the SARS-CoV-2 S protein). On 19 March 2021, Germany reported 13 cases of sinus or cerebral vein thrombosis for more than 1.6 million AZD1222 Oxford–AstraZeneca vaccines administered, with some of these patients having a heparin-induced thrombocytopenia (HIT)-like syndrome. Rare cases of arterial thromboembolism were reported associated with the BNT162b2 mRNA (Pfizer-BioNTech), without evidence of thrombocytopenia [15].

**Figure 3:**
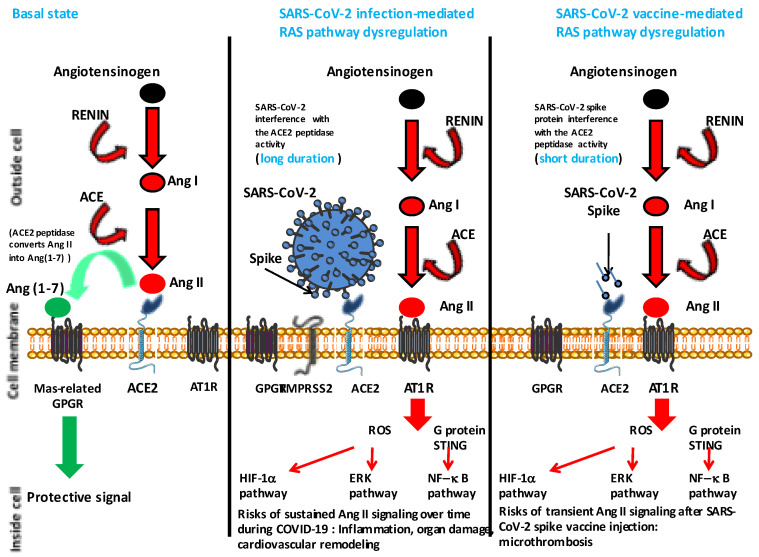
Simplified diagram of the renin-angiotensin system (RAS) in normal conditions and under dysregulation mediated either by the SARS-CoV-2 or a SARS-CoV-2 spike based vaccine. The left panel simply indicates that ACE2 converts Ang II to Ang (1-7) leading to a protective signal (a balance between Ang I and Ang (1-7) is required for homeostasis). The middle panel illustrates the possible dysfunction of signals when SARS-CoV-2 is attached to its ACE2 receptor (this dysfunction has been experimentally and clinically proven). Under this condition the balance between Ang (1-7) and Ang II is in favor of Ang II. Ang II accumulates and binds to AT1R, leading to proinflammatory and hypoxia signals that can trigger tissue damage (in particular to the lung and heart). The right panel illustrates a hypothetical transient dysfunction of the RAS pathway when an excess of SARS-CoV-2 spike protein is present in tissues, as a consequence of recent vaccination, leading to a short period during which there is a risk of micro-thrombosis. The spike protein (vaccine) is likely to mimic the virus by interacting with the cellular ACE2; however in that case the process is expected to be of short duration in contrast to the process mediated by the virus that continues for as long as the virus replicates in a patient. Angiotensin I = Ang I; angiotensin II = Ang II; angiotensin (1-7) = Ang (1-7); angiotensin I converting enzyme = ACE; angiotensin I converting enzyme 2 = ACE2; angiotensin II type 1 receptor = AT1R; G protein-coupled receptor = GPGR; reactive oxygen species = ROS; hypoxia inducible factor 1 alpha = HIF-1α; extracellular signal regulated kinases = ERK; nuclear factor kappa B = NF-B.

It has been considered that one apparent difference between adenoviral vector-based vaccines and RNA-based vaccines is that beside the adverse effects of cerebral venous sinus thromboses (CVST) reported with both types of vaccines, splanchnic vein thromboses (SVT) and other thromboembolic events seemed to be specific of adenoviral vector-based vaccines [57]. Kowarz and colleagues [54] showed that DNA-encoded mRNA coding for the spike protein can be spliced (the cloned cDNA exhibits potential splice donor and splice acceptor cryptic splice sites) in a way that the transmembrane anchor of spike is lost, and a nearly full-length spike is secreted from infected cells capable of binding the ACE2 receptor. They found significant amounts of splice events using genome-wide RNA Seq data of ChAdOx1-transduced cells and concluded that such vaccines can sometimes induce a COVID-19 mimicry syndrome involving ACE2, soluble spike, anti-spike antibodies and mechanisms of antibody-dependent cell cytotoxicity (ADCC) and complement-dependent cytotoxicity (CDC), both mechanisms acting on thrombus formation. Although this could be an explanation of the post-vaccine pathophysiological dysfunctions, we suggest a simpler hypothesis also based on mimicry that could account for both rare myocarditis and thrombosis events reported post-vaccine against SARS-CoV-2 and postulate that interaction between the viral spike and ACE2 reduces ACE2 protease activity and increases Ang II, leading to lung cells hypoxia and dysfunction in the RAS pathway (thrombosis) (Figure 3). This is simply an extension of the model we proposed early in the pandemic to explain the thrombotic events induced by the virus during COVID-19 [18].

In the study by Welsh and colleagues on people who received COVID mRNA vaccine, 15 cases of thrombocytopenia were identified among 18,841,309 doses of the Pfizer-BioNTech COVID-19 vaccine, and 13 cases of thrombocytopenia were identified among 16,260,102 doses of the Moderna COVID-19 vaccine suggesting a risk of 0.8/100,000 [58]. This rare complication, referred to as SARS-CoV-2 vaccine-induced thrombotic thrombocytopenia (VITT), involves the induction of auto-antibodies to the platelet factor 4 (PF4; a small cytokine belonging to the CXC chemokine family), rather than the ACE2 pathway.

## 5. SARS-CoV-2 Vaccination-Induced Thrombotic Thrombocytopenia (VITT)

Thrombosis is one of the most severe adverse consequence of vaccines. In the case of the Oxford–AstraZeneca SARS-CoV-2 adenovirus vaccine and the Moderna SARS-CoV-2 mRNA vaccine, evidence of a vaccine-induced thrombosis event with a platelet count decrease of more than 50% and a procoagulant state with secondary development of thrombotic events, the highest frequency of which are in aortic vessels, aorto-iliac site, basivertebral veins, cerebral venous sinus, deep veins, jugular veins and hemiazygos vein were reported [59]. A study conducted in the UK between March 22 and June 6, 2021 reported 50 probable cases of VITT in patients (median age 48 years) with no identifiable medical risk factors who had received the first dose of ChAdOx1 nCov-19 AstraZeneca [60].

A clinical study investigating adverse events (thrombosis and thrombocytopenia) in 23 patients who received a first dose of the ChAdOx1 nCoV-19 vaccine reported induction of auto-antibodies to PF4 [61]. Of the 22 patients who presented with acute atypical thrombosis, 13 presented cerebral venous thrombosis, 4 presented a pulmonary embolism, 1 presented deep venous thrombosis and bilateral adrenal hemorrhage, 2 presented an ischemic stroke affecting the middle cerebral artery, and 2 presented portal vein thrombosis. Another patient presented with isolated thrombocytopenia and a hemorrhagic phenotype. Twenty-two of these patients tested positive for anti-PF4 auto-antibodies, which could trigger platelet activation and coagulopathy. A similar observation was reported by other clinicians [12]. Indeed, the platelet Fc receptor can bind to the complex formed between heparin-PF4 and anti-PF4 auto-antibodies, and the cross-linking of FcγRIIa (CD32a) receptors at the membrane of platelets induces platelet activation and aggregation [62,63].

A study across the UK, performed between 1 April and 20 May 2021, reported on 70 cases of VITT and found that VITT-associated cerebral venous thrombosis symptoms had more intracranial veins thrombosed than non-VITT patients [64]. This rare complication was found to occur in approximately 2/100,000 to 1/100,000 recipients of the adenovirus-based COVID-19 vaccines produced by Oxford–AstraZeneca or Johnson & Johnson companies [13,65,66,67]. Notably, in one series of 220 patients with characterized or probable VITT patients who were exposed to heparin (early in the COVID-19 pandemic), they were found to have no worse clinical outcomes than those who had never been treated with heparin [60].

**Figure 4:**
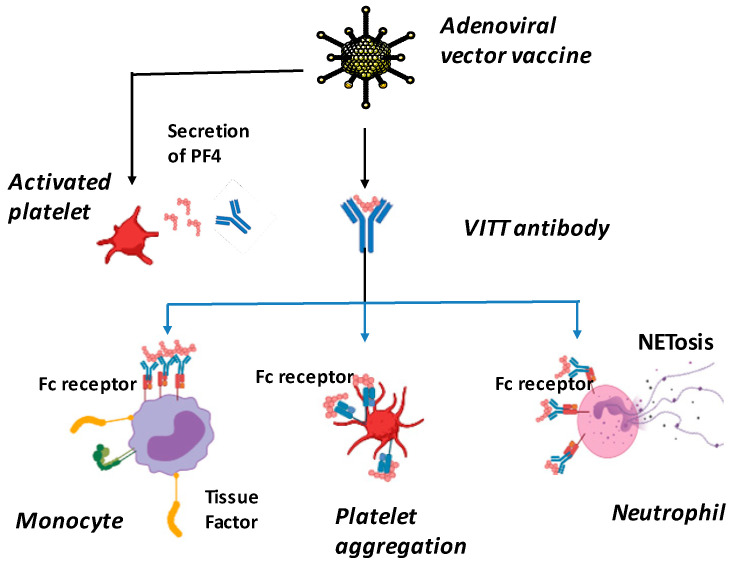
Proposed mechanisms of VITT pathogenesis. Anti-PF4 antibodies induce platelet aggregation, neutrophil and monocyte activation that activate coagulation and inflammation, and ultimately progresses to thrombosis. Extracellular neutrophil traps (NETs); platelet factor 4 (PF4). NETosis, or NET activation and release, is a dynamic process that can come in two forms, suicidal and vital NETosis. Overall, many of the key components of the process are similar for both types of NETosis.

VITT is characterized by the occurrence of thrombosis in an unusual location, such as cerebral venous sinuses, thrombocytopenia of splanchnic veins, decreased fibrinogen levels and elevated D-dimer levels. These events occur in males or females, generally between the ages of 18 and 79, between 4 and 48 days after vaccination with an adenovirus-based vaccine such as Oxford–AstraZeneca (ChadOx1 nCov-19) or Janssen/Johnson & Johnson (Ad26.COV2.S) [13,68]. Vaccine components, including adenovirus hexonal protein (the most abundant of the structural proteins in the icosahedral adenovirus capsid) and/or adenovirus, can generate neoantigenic complexes with PF4, released from platelet α-granules. These PF4/vaccine neoantigens, combined with vaccine-induced inflammation, stimulate B cells to produce anti-PF4 antibodies. FcγIIa receptors on the platelet surface bind to the low-affinity Fc domain of IgG in a mechanism that activates platelets and causes thrombosis and thrombocytopenia. Anti-PF4/vaccine antibodies induce cellular activation of hemostasis involving platelets, neutrophils and monocytes. Neutrophil activation induces a NETosis process, which contributes to the activation of coagulation cascades. Monocyte activation is responsible for tissue factor (TF) release (Figure 4). The presence of anti-PF4 antibodies by ELISA is found in most patients.

A diagnosis of VITT should include the following criteria:

(i). A COVID-19 vaccine administered between 4 and 42 days before the onset of symptoms;
(ii). Venous or arterial thrombosis that may occur in uncommon sites such as cerebral and splanchnic vessels;
(iii). Thrombocytopenia;
(iv). A positive PF4 HIT ELISA result (non-rapid tests should be used);
(v). Elevated D-dimer levels.

The use of non-heparin anticoagulants such as direct thrombin inhibitors, factor Xa inhibitors and direct oral anticoagulants (DOACs) has been suggested [67,69].

In addition, it was previously found that the platelet activation ability can be neutralized in the presence of high heparin concentrations and by the use of monoclonal antibodies binding the CD32a [70].

## 6. Incidence Rates of Acute Myocardial Infarction, Deep Vein Thrombosis, Pulmonary Embolism, Myocarditis and Pericarditis Following COVID-19 Vaccine

This is a very difficult and very important question to be answered. After the first alerts from the medical community regarding possible adverse effects of COVID-19 vaccination, many countries have attempted to set up enhanced surveillance to record adverse events and evaluate COVID-19 vaccine safety. However, the incidence rates varied greatly between databases. The multiple press release of preliminary information regarding warning signals triggered a huge debate over COVID-19 vaccine safety, both at the level of medical experts, politicians and the world population, some of whom have shown growing hostility to vaccination.

To date, the most documented study on this topic has been reported by Li and colleagues [71] who have tried to quantify the background incidence rates of 15 pre-specified adverse events of special interest (AESIs) associated with COVID-19 vaccines. They found considerable heterogeneity between geographies, vaccine formulation and databases and suggest caution when interpreting the differences between clinically observed and expected rates according to vaccine surveillance. The AESIs definition as well as the data model may be different from country to country, showing the need for standardization for safety surveillance. Considerable variability was also found between age group and sex, as illustrated in Table 2, which was designed according to data published by Li and colleagues. The incidence of each AESI depends mainly on the type of vaccine formulation and the age of the patients. There is certainly a paradox. Indeed, some cardiovascular side effects increase with age, but elderly patients are very often treated long-term to prevent these pathologies. Thus, the incidence rates recorded are probably underestimated. Moreover, it is not possible to establish the incidence rate of hypertensive episodes, because the studies use different criteria to evoke the notion of hypertension. According to Kim and colleagues [72], COVID-19 mRNA vaccines have a much higher risk for hypertensive crisis (adjusted odds ratio 12.72, 95% CI 2.47–65.54).

## 7. Discussion

Every infectious disease is a new pathophysiological problem in itself, and the development of a vaccine presents a new challenge. Depending on the pathogen, vaccines have shown marvelous success in controlling some infectious diseases, while, in contrast, vaccines have totally failed to control other infectious pathogens. During SARS-CoV-2, both B and T cell responses are elicited. Regarding the humoral response, the dominant antibody response is against the conserved N protein and the more variable S protein, which is the target for neutralizing activities. It was often observed that healed patients display high titers of SARS-CoV-2 neutralizing antibodies, suggesting that inducing an immune response against the spike, particularly the RBD, is an appropriate strategy for developing a vaccine. Indeed the induction of neutralizing antibodies has been widely documented [73,74,75], albeit with less efficacy on the SARS-CoV-2 Omicron sublineages [76,77]. Moreover, increased memory B cell potency was reported following SARS-CoV-2 mRNA booster injections [78]. However, there is a poor correlation between antibody levels and the frequency of memory B cells generated during immune response to SARS-CoV-2 antigens [79,80]. Moreover, SARS-CoV-2 is a respiratory tract pathogen and data on the role of mucosal immunity with anti-SARS-CoV-2 IgA are scarce. In addition, protection against SARS-CoV-2 is likely to require the induction of CD4+ memory T cells and CD8+ cytotoxic T lymphocytes responses [81,82,83].

The induction of an appropriate immune response is a central objective of a vaccine, but other considerations should be taken into account when designing a vaccine. Protection against virus entry into cells is one of the main objectives, which leads vaccine manufacturers to include the viral envelope (often exclusively) in their vaccine formulation with the aim of inducing the production of neutralizing antibodies in the vaccinated person. However, it is worth considering the risks of undesired effects, such as the antibody-dependent enhancement (ADE) of infection by the Fc receptor, which is one of the major banes of successful vaccination [8,84]. The amino acid sequences supporting the induction of antibodies involved in ADE could be removed during vaccine design, as has been suggested [85]. Among other problems to be taken into account is the physiological function of the virus receptor expressed at the cell surface. A different example would involve imagining that we want to develop a vaccine against the human immunodeficiency virus (HIV), another virus which also represents a global challenge. The immunological rationale would tend to also use the envelope protein to induce protective immunity, an apparently reasonable approach. However, if once in contact with this envelope protein, the virus receptors (CD4 and CXCR4) induce intracellular signal leading to cell apoptosis, then questions must be asked about the advisability of using the HIV envelope glycoprotein without modifying it, because the risk would be to induce the very CD4+ lymphopenia that the vaccine is supposed to prevent [86,87]. Although SARS-CoV-2 used a different receptor from that used for HIV entry, the same question arises with the SARS-CoV-2 receptor, ACE2. Before being regarded as the SARS-CoV-2 receptor, ACE2 was known to play a central role in the RAS, which regulates cardiovascular and renal functions and maintains BP homeostasis as well as fluid and salt balance. An uncontrolled RAS pathway may lead to the development of vascular remodeling and vascular rigidity, which may predispose the individual to left ventricular hypertrophy of the heart and fibrosis, acute kidney injury and extensive microthrombosis in coronary and pulmonary circulation [88,89]. Ang II is the main harmful effector molecule synthesized in excess in situations of RAS imbalance [19,90]. Among its other effects, ACE2 is associated with vasoconstriction and hypertension, and it accelerates thrombosis in arterioles by activating the coagulation cascade and the platelet-derived growth factor [91,92]. The multiple effects of Ang II are mediated through its binding to Ang II type I and type II receptors (AT1R and AT2R, respectively) expressed in arterioles and several organs, including the kidney, pancreas, heart and the brain [93]. The interaction between Ang II and AT1R enhances oxidative injury by reactive oxygen species (ROS) and endothelial injury by inhibiting nitric oxide (NO) synthesis [94,95,96]. Ang II activates the flow of neutrophils and macrophages to the affected tissues and inhibits the production of NO, leading to vascular injury [97]. Moreover, Ang II also induces several signaling pathways, including G-protein-coupled receptors, protein kinase C, serine/threonine kinase, serine tyrosine kinases and ERK/JNK activation, leading to proinflammatory responses characterized by the synthesis of IL-6, TNFα and other cytokines [98,99]. We had previously hypothesized that, by binding to ACE2, SARS-CoV-2 causes a dysfunction of this molecule, the consequence of which is the accumulation of Ang II and an activation of intracellular signals via AT1R, which are responsible for harmful events at the level of cell, tissues and organs to the point of being a major element of the physiopathology of COVID-19 [18]. Our investigation into the RAS pathway in a cohort of COVID-19 patients has confirmed the validity of this model [100].

Insofar as this effect of the virus is mainly linked to the interaction between the spike of SARS-CoV-2 and the enzymatic domain of ACE2 usually responsible for the cleavage of Ang II into angiotensin 1-7, it is very likely that vaccines based on the envelope protein of the virus induce virus-like effects (mimicry) via binding to ACE2 and leading to extremely rare but severe thrombotic events. Apart from ACE2, other carboxypeptidases can directly catalyze the formation of Ang 1-7 from Ang II: the prolyl carboxypeptidases (PRCP) and the prolyl oligopeptidase (POP) [101,102]. After observing that the increased catalytic activity of POP and PRCP is not typical in the young, but more pronounced in elderly individuals with comorbidities or previous cardiovascular events, Angeli and colleagues [103] have hypothesized that the adverse reactions to COVID-19 vaccination associated with Ang II accumulation (that they named “Spike effect” of COVID-19 vaccines), are reasonably expected to be more common in younger and healthy subjects. COVID-19 mRNA vaccine’s adverse reactions figures accrued so far may be somewhat underestimated due to the fact that a considerable proportion of elderly people are already under cardiovascular therapies (due to cardiovascular morbidities), which can thus prevent or reduce the manifestation of vascular and coagulation dysfunction induced by the vaccine. In addition, it cannot be ruled out that indirect effects, such as alcohol consumption, could have increased the risk of adverse reactions to COVID-19 vaccination. The consumption of alcoholic beverages increased during lockdowns and remained at high levels during the peak of vaccination, especially among young people. It has been postulated that alcohol consumption, known to affect expression of ACE2, may have contributed to increasing the “Spike effect” risk of COVID-19 vaccines [104]. Finally, if ACE2 and Ang II clearly play a role in the thrombosis and cardiovascular complications following the COVID-19 mRNA vaccine, it has also been suggested that another physiopathological mechanism can sometimes trigger the cerebral venous sinus thrombosis (CVST) and cerebral venous thrombosis observed after vaccination with an adenoviral-vector-based vaccine [105]. In these ACE2 and Ang II-independent thrombotic adverse reactions in which thrombocytopenia is almost universal (VITT), the reaction is associated with circulating anti-PF4 antibodies, which generate procoagulant platelets and stimulates NETosis. The DNA released by NETosis amplifies the immune injury [106,107], and activates complement, which is deposited on the endothelium. The endothelial cells become activated, express tissue factor and release von Willebrand factor (VWF). VWF binds to PF4 and subsequently to anti-PF4 antibodies, which in turn activates neutrophils and propagates thrombin generation. Thus, endothelial activation likely participates in the occurrence of thrombotic events notably through the expression of tissue factor [108,109,110]. Fortunately, VITT is very rare.

Notably, routine thromboprophylaxis of SARS-CoV-2-infected patients was found to reduce the length of the hospitalization and mortality [111,112,113,114]. Moreover, it has been suggested that patients with isolated thrombocytopenia who have not experienced a thrombotic complication may be treated with a direct oral anticoagulant (DOAC) to help prevent thrombotic complication [67]. In this context, mutations could be introduced in the spike gene construct of the vaccine to prevent this adverse effect on ACE2 function. However, it is obvious that this strategy would have a high probability of altering the immune response to the spike RBD, which is the main target of neutralizing antibodies. Alternatively, it would no doubt be wise to use a short (48 h) anticoagulant therapy aimed to prevent the possible thrombotic complication at the time when SARS-CoV-2 vaccine is injected, particularly in people at risk of thrombosis.

## Figures and Tables

**Table 1:** Harmful effects of COVID-19 vaccine.

Study	Source	Cohort (*n*)	Year	Vaccine	Outcome	
					Characteristic	N, %
Bouhanick et al. [42]	Pharmacovigilance database	91,761	2021	BNT162b2, ChAdOx1nCoV-19, Ad26.COV2.S	Abnormal BP *	1776, 1.9%
Kaur et al. [44]	Pharmacovigilance database	30,523	2021	BNT162b2 ChAdOx1nCoV-19mRNA-1273 BNT 62b2	Abnormal BP * Stage III hypertension or hypertensive emergency	283, 5.82% 36, 0.11%
Lehmann et al. [43]	Pharmacovigilance database	212,053	2021	ChAdOx1nCoV-19, Ad26.COV2.S mRNA-1273	Abnormal BP * Stage III hypertension or hypertensive emergency	6130, 2.9% 551, 0.25%
Tran et al. [49]	Cross-sectional online survey	1028	2021	ChAdOx1nCoV-19	Self-reported hypertension	52, 5%
Zappa et al. [40]	Cross-sectional online survey	113	2021	BNT162b2	Raise in home BP > 110 mmHg Stage III hypertension or hypertensive emergency	6, 5.3% 2, 1.7%
Bouhanick et al. [50]	Patients and healthcare workers	21,909	2022	BNT162b2	Persistent BP (≥140/90, 15 min after vaccination) Stage III hypertension or hypertensive emergency	5197, 23.7% 709, 3.23%

* Abnormal BP refers to increase in blood pressure values. It is important to underline the heterogeneity of the studies regarding the time interval elapsed in relation to the moment of vaccination, the measurement techniques (self-measurement or medical surveillance), the absence of precise data on the history of hypertension and/or the co-morbidities of patients.

**Table 2:** Summary of pooled estimated age and sex stratified incidence rates per 100,000 person years (95% prediction intervals), according to meta-analyses reported by Li and colleagues [71].

Mean of Incidence Rate/100,000 Person-Years	Acute Myocardial Infarction		Deep Vein Thrombosis		Pulmonary Embolism		Myocarditis, Pericarditis	
Years	Men	Women	Men	Women	Men	Women	Men	Women
18–34	16	6	80	140	20	38	37	16
35–54	172	54	272	306	81	80	37	22
55–64	171	467	499	428	171	125	45	31
65–74	653	312	695	683	256	217	49	35
75–84	934	617	831	975	349	358	54	39
>85	1514	1144	1003	1206	398	427	41	34

The incidence rates recorded for the elderly patients are probably underestimated due to the fact that a considerable proportion of elderly people are already under cardiovascular therapies.
